# Supplementary material for: Modeling glioblastoma heterogeneity as a dynamic network of cell states
Source: Mol Syst Biol. 2021 Sep 16;17(9):e10105. doi: 10.15252/msb.202010105 (PMC8444284; doi:10.15252/msb.202010105)
Supplement: Supplementary file 6 — Source Data for Figure 5 [file MSB-17-e10105-s004.zip › Figure5A_sourcedata/GSEA_3017/nosigdb_stateB.GseaPreranked.1622795021725/DOROSZKO_U3054MG_CLUSTER_8.html]

Details for gene set DOROSZKO\_U3054MG\_CLUSTER\_8[GSEA]

|  || Dataset | state43017 |
| Phenotype | NoPhenotypeAvailable |
| Upregulated in class | na\_neg |
| GeneSet | DOROSZKO\_U3054MG\_CLUSTER\_8 |
| Enrichment Score (ES) | -0.3300502 |
| Normalized Enrichment Score (NES) | -2.181599 |
| Nominal p-value | 0.0 |
| FDR q-value | 0.0064137406 |
| FWER p-Value | 0.014 |
Table: GSEA Results Summary

  

Fig 1: Enrichment plot: DOROSZKO\_U3054MG\_CLUSTER\_8      
 Profile of the Running ES Score & Positions of GeneSet Members on the Rank Ordered List

  

| PROBE | GENE SYMBOL | GENE\_TITLE | RANK IN GENE LIST | RANK METRIC SCORE | RUNNING ES | CORE ENRICHMENT || 1 | CCP110 |  |  | 204 | 0.411 | -0.2315 | No |
| 2 | PCM1 |  |  | 232 | 0.393 | -0.2236 | No |
| 3 | PKP4 |  |  | 311 | 0.342 | -0.2911 | Yes |
| 4 | LMF2 |  |  | 338 | 0.330 | -0.2890 | Yes |
| 5 | MACF1 |  |  | 342 | 0.328 | -0.2557 | Yes |
| 6 | TACC1 |  |  | 359 | 0.322 | -0.2409 | Yes |
| 7 | ACTN4 |  |  | 376 | 0.316 | -0.2267 | Yes |
| 8 | CEP192 |  |  | 395 | 0.310 | -0.2160 | Yes |
| 9 | SMC5 |  |  | 420 | 0.300 | -0.2146 | Yes |
| 10 | AHI1 |  |  | 425 | 0.299 | -0.1861 | Yes |
| 11 | DYRK1A |  |  | 450 | 0.293 | -0.1854 | Yes |
| 12 | SYNE1 |  |  | 458 | 0.291 | -0.1619 | Yes |
| 13 | RBMS1 |  |  | 500 | 0.281 | -0.1858 | Yes |
| 14 | MBNL2 |  |  | 517 | 0.278 | -0.1760 | Yes |
| 15 | SCAPER |  |  | 553 | 0.272 | -0.1927 | Yes |
| 16 | VPS13A |  |  | 579 | 0.268 | -0.1963 | Yes |
| 17 | IFRD1 |  |  | 594 | 0.264 | -0.1854 | Yes |
| 18 | GNA11 |  |  | 596 | 0.264 | -0.1567 | Yes |
| 19 | PUM2 |  |  | 613 | 0.261 | -0.1488 | Yes |
| 20 | ICA1L |  |  | 621 | 0.259 | -0.1289 | Yes |
| 21 | UCHL5 |  |  | 671 | 0.252 | -0.1670 | Yes |
| 22 | CHD7 |  |  | 706 | -0.284 | -0.1810 | Yes |
| 23 | NEAT1 |  |  | 714 | -0.308 | -0.1554 | Yes |
| 24 | IDI1 |  |  | 734 | -0.397 | -0.1362 | Yes |
| 25 | DDX21 |  |  | 745 | -0.440 | -0.0997 | Yes |
| 26 | SNHG25 |  |  | 746 | -0.444 | -0.0492 | Yes |
| 27 | SOX11 |  |  | 754 | -0.576 | 0.0068 | Yes |
Table: GSEA details [plain text format]

  

Fig 2: DOROSZKO\_U3054MG\_CLUSTER\_8: Random ES distribution      
 Gene set null distribution of ES for **DOROSZKO\_U3054MG\_CLUSTER\_8**
